# Impact of vitamin D on maternal and fetal health: A review

**DOI:** 10.1002/fsn3.2948

**Published:** 2022-07-07

**Authors:** Rizwan Arshad, Aysha Sameen, Mian Anjum Murtaza, Hafiz Rizwan Sharif, Iahtisham‐Ul‐Haq, Sahifa Dawood, Zahoor Ahmed, Arash Nemat, Muhammad Faisal Manzoor

**Affiliations:** ^1^ University Institute of Diet and Nutritional Sciences The University of Lahore Gujrat Campus Gujrat Pakistan; ^2^ National Institute of Food Science and Technology University of Agriculture Faisalabad Faisalabad Pakistan; ^3^ Institute of Food Science and Nutrition University of Sargodha Sargodha Pakistan; ^4^ Kausar Abdullah Malik School of Life Sciences Forman Christian College University Lahore Pakistan; ^5^ Human Nutrition and Dietetics School of Food and Agricultural Sciences, University of Management and Technology Lahore Pakistan; ^6^ Department of Microbiology Kabul University of Medical Sciences Afghanistan; ^7^ School of Food and Biological Engineering Jiangsu University Zhenjiang Jiangsu China

**Keywords:** birth defects, calcitriol, fetal growth, maternal health, vitamin D

## Abstract

The role of vitamin D in improving maternal health and reducing the risk of developmental disorders in fetus has been an important domain of research since the past few years. Vitamin D, owing to its immunomodulatory, anti‐inflammatory, developmental roles, and regulating calcium homeostasis, is predicted to have a significant influence on maternal and fetal health status. Several observational studies and clinical trials, determining the impact of vitamin D on gestational diabetes, C‐section, postpartum depression, pre‐eclampsia, miscarriages, and preterm delivery, have been elaborated in this review. In addition, fetal birth defects including neurological development, reduced birth weight, respiratory infections, bone development, and altered anthropometrics have also been summarized with available evidences. Other important mechanisms related to the roles of vitamin D in the body are also explained. Furthermore, recent studies determining the effect of vitamin D at genetic level will also help in understanding and future design of research in the area of maternal and fetal health.

## Graphical abstract

The role of vitamin D in improving maternal health and reducing the risk of developmental disorders in fetus, has been an important domain of research since past few years. Vitamin D, owing to its immunomodulatory, anti‐inflammatory, developmental roles and regulating calcium homeostasis, is predicted to have significant influence on maternal and fetal health status. Several observational studies and clinical trials, determining the impact of vitamin D on gestational diabetes, C‐section, postpartum depression, pre‐eclampsia, miscarriages and preterm delivery, have been elaborated in this review.

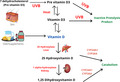

## 1 INTRODUCTION

Vitamin D, a group of fat‐soluble secosteroids, is considered a vital prohormone in the body. In addition to regulating the homeostasis of calcium, magnesium, and phosphorus for bone formation, it plays a significant role in different developmental progressions, especially in neurological and cardiovascular processes, regulation of innate and acquired immunity, and reproductive health (Arshad et al., 2021; Manzoor et al., 2021; Yan et al., 2016). Maternal vitamin D status during conception, gestational period, and perinatal stages aids in regulating embryogenesis, skeletal development, and calcium level of the growing fetus (Wagner et al., 2017).

In the past few years, vitamin D malnutrition has become prevalent among populations of different countries, races, and age groups, especially among women of reproductive age (WRA) (Shah et al., 2021; Traglia et al., 2020). Factors associated with vitamin D insufficiency are substantial use of sun protection beauty products, insufficient exposure to sunlight, use of tobacco, obesity, insufficient vitamin D intake or intestinal malabsorption, seasonal variation that is observed at temperate latitudes, and some pathological conditions like kidney or liver failure, chronic inflammation, and use of contemporary medications (Anwar et al., 2021; Palacios et al., 2016; Weishaar et al., 2016). It has been recently observed that hypovitaminosis D during pregnancy is emerging as an important global issue and it can affect the programming of the next generation which suggests that the negative effects of deficiency of this vitamin may considerably affect the progenies (Larqué et al., 2018), which suggests that the negative effects of deficiency of this vitamin may considerably affect the progenies. The objective of this review is to highlight the role of vitamin D as immunomodulatory, anti‐inflammatory, and in improving maternal health and reducing the risk of developmental disorders in fetus.

## 2 PHYSIOLOGY AND RECOMMENDATIONS OF VITAMIN D

In the human body, vitamin D exists in two forms: vitamin D2 (Ergocalciferol) and vitamin D3 (Cholecalciferol). Major sources of vitamin D2 are plants and vegetables along with fatty fish, eggs, and mushrooms, whereas most of vitamin D3 is synthesized within the body. Dietary supplements of these both are available, where calcitriol (supplemental form of vitamin D3) is more common (Larqué et al., 2018).

**FIGURE 1:**
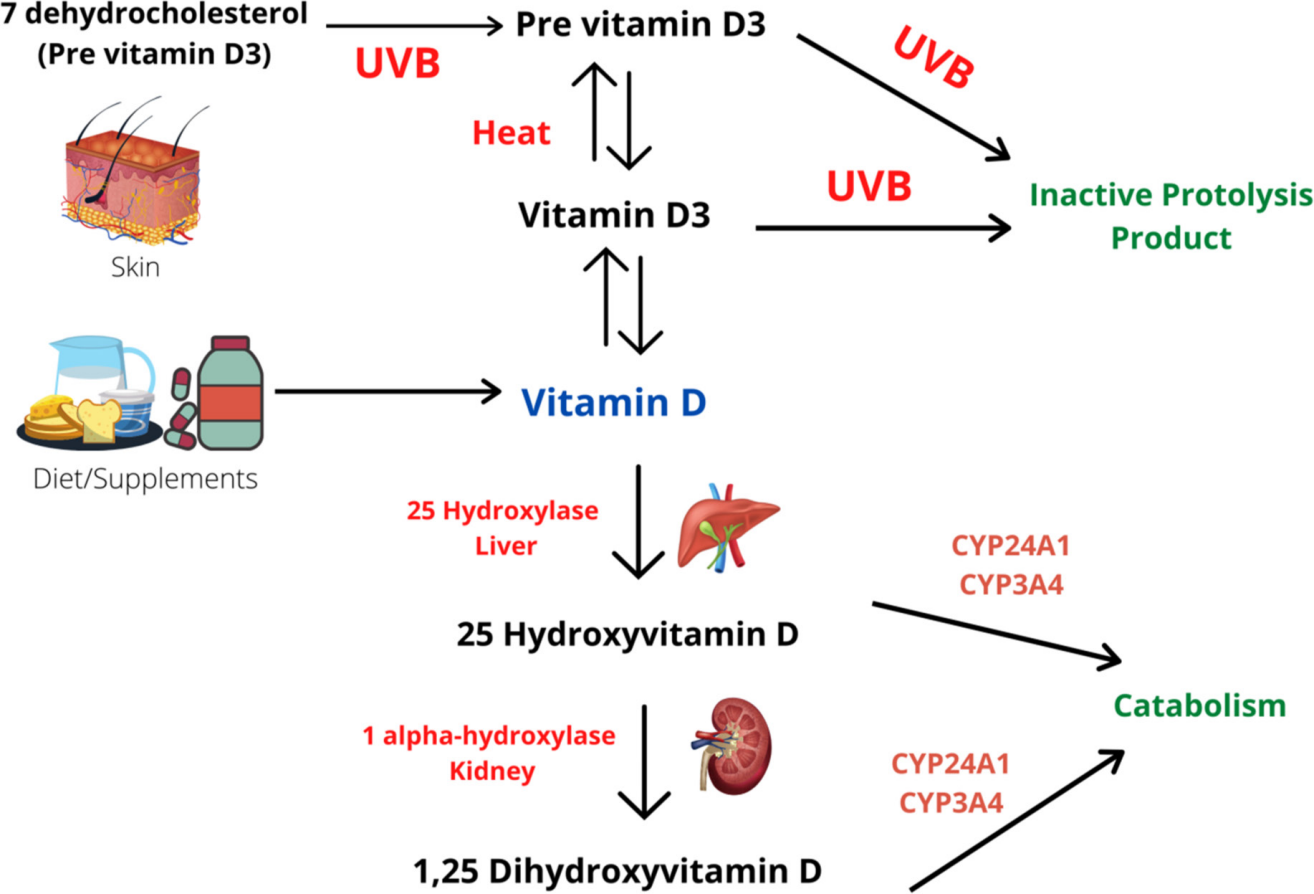
Mechanism for the activation of vitamin D

Vitamin D2 is converted to inactive metabolite 25‐hydroxyvitamin D (25 [OH]D) by cytochrome P450 enzyme 25‐hydroxylase (CYP27A1) in the liver. It is then activated in kidneys by the enzyme 25(OH)D‐1α‐hydroxylase (CYP27B1) into 1,25(OH)2D (Palacios et al., 2016). Endogenous production of vitamin D3(25(OH)D3) occurs via isomerization of 7‐dehydrocholesterol (DHCR7) in skin cells, during sun exposure, by ultraviolet B rays (UVB) having a spectral wavelength from 29 to 315 nm. Studies indicated extrarenal activity of 1α‐hydroxylase enzyme in placental, skin, and skeletal tissues, which elucidate an enhanced production of vitamin D3 which then diffuses in the blood where it binds to vitamin D binding protein (VDBP) and lipoproteins. The most abundant form of vitamin D, found circulating in the blood, is 25(OH)D. The synthesis of vitamin D and its availability in the body are controlled by parathyroid hormone (PTH) and concentration of calcium and phosphorus in blood (Ramasamy, 2020) (Figure 1).

Keeping the fetal needs in consideration, the Institute of Medicine (IOM) and the European Calcified Curtis et al. (2018) recommended a daily intake of 600 IU of vitamin D during gestational and lactation period. 25(OH)D is considered the biomarker for the status of vitamin D in the body (Cashman et al., 2017). According to Flood‐Nichols et al. (2015), vitamin D status can be categorized in the following ranges: optimal [≥30 ng/ml (75 nmol/L)], insufficient [21–29 ng/ml (51–74 nmol/L)], deficient [<20 ng/ml (50 nmol/L)], and severely deficient [<10 ng/ml (25 nmol/L)].

## 3 METABOLISM OF VITAMIN D IN PLACENTA

**FIGURE 2:**
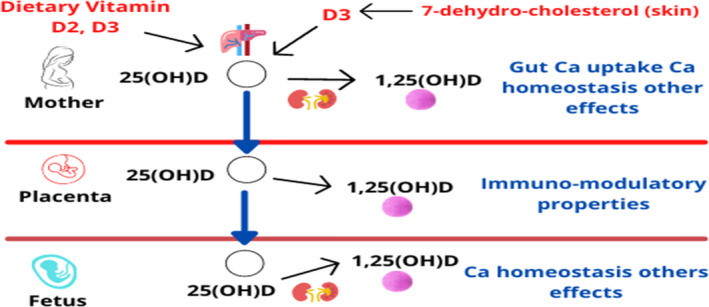
Effects of vitamin D during pregnancy

Gestational period includes a number of physiological changes in mother's body to achieve a healthy, uncomplicated delivery, and provide an optimum intrauterine environment for the growing fetus (Simner et al., 2020). During pregnancy, vitamin D homeostasis is regularized by three adaptations including increase in maternal calcitriol, VDBP concentrations, and adequate availability of maternal 25(OH)D. These variations are evident at systemic and placental circulation levels, suggesting that the placenta is a main site for vitamin D metabolism (Wagner et al., 2017) (Figure 2).

**FIGURE 3:**
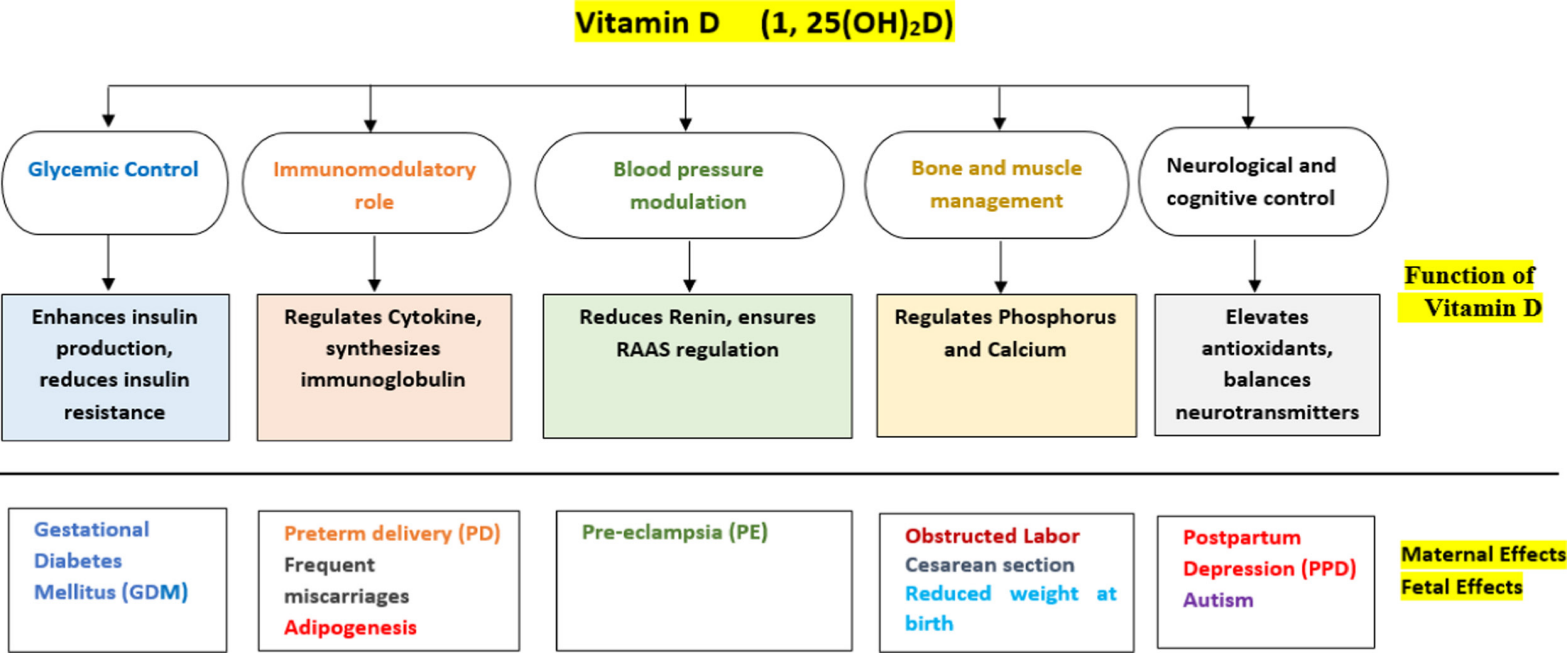
Role of vitamin D on maternal and fetal health

Along with the transmission of oxygen and nutrients, the placenta also mediates the immune‐‐tolerance adaptation during pregnancy. Fetus cannot synthesize its own vitamin D (calciferol), therefore maternal vitamin D or its other biological metabolites must be transferred to the fetus through placenta. It is not transferred in its activated form, i.e., 1,25(OH)2D, through placental tissue, rather as the inactivated precursor form, 25(OH)D, which crosses the placental barrier to the fetal compartment (Liu & Hewison, 2012). The placenta contains the enzyme 1‐α‐hydroxylase that can possibly activate 25(OH)D‐producing 1,25(OH)2D (Workalemahu et al., 2017). Moreover, placenta can also convert 25(OH)D by 24‐hydroxylation to inactivated 24,25(OH)2D, which has paracrine control for metabolism and modulation of vitamin D status, thus moderating its anti‐inflammatory effects and maternal–perinatal developmental outcomes (Larqué et al., 2018). By the second trimester, serum concentration of 1,25(OH)2D increases to double as compared to nongravid stage, and the concentration further increases continuously reaching up to two to three times as that of nonpregnant women. It has been noticed that the concentration of 1,25(OH)2D in cord blood is closely related to fetal concentration of 25(OH)D and this concentration is independent of the general calcium‐regulated mechanism (Wagner et al., 2017). However, the mechanism of metabolism and transmission of vitamin D through the placenta and the influence of vitamin D on placental tissues are still not fully understood (Simner et al., 2020) (Figure 3).

## 4 MATERNAL VITAMIN D DEFICIENCY AND ITS EFFECTS ON HER HEALTH

### 4.1 Gestational diabetes

Gestational diabetes mellitus (GDM) is a condition identified as hyperglycemia during the second or third trimester of pregnancy. If a woman appears positive for diabetes mellitus in the first trimester, then she must have pre‐existing diabetes, either type 1 or type 2 (Haq et al., 2021; Lacroix et al., 2014).

Several studies provide sufficient rationale for the functions of vitamin D in glucose homeostasis. Possible mechanisms that show the interrelation between the alteration in glucose metabolism and vitamin D status is (1) activated vitamin D attaches to vitamin D receptors (VDR) on pancreatic β‐cells; (2) 1‐α‐hydroxylase enzyme expresses in pancreatic β‐cells; (3) extracellular calcium is regulated through the pancreatic β‐cell which in return controls insulin sensitivity and secretion; and (4) vitamin response elements are present in insulin gene promoter. As insulin sensitivity improves in adipose, skeletal, and liver muscle cells, it initiates the activity of insulin receptor and affects systemic inflammation, which is carried out by cytokines affecting the function of β‐cells (Ateş et al., 2017). Thus, any alteration of vitamin D receptors or vitamin D insufficiency may lead to diabetes, but its effect in GDM still remains questionable.

Keeping in view the changing maternal concentration of vitamin D during different trimesters, some of the following studies explain the association. According to Lacroix et al. (2014), maternal vitamin D deficiency during first trimester surges the chances for GDM development. Later, another study comparing maternal vitamin D status during first trimester of women with GDM and non‐GDM found no significant association. In this study, women who developed the symptoms of GDM and healthy group of controls, both were found as severe vitamin D deficient (<10 ng/ml) during their first trimester of pregnancy (Ateş et al., 2017). For second and third trimester of pregnancy, maternal vitamin D deficiency is evident to elevate the chances for GDM. Results of a recent research showed that majority of GDM women were mildly to moderately deficient of vitamin D (>25 but <75 nmol/L)(Rajput et al., 2019). Thus, it is essential to normalize vitamin D intake in last two trimesters to avoid such morbidities. Aiding these results, a meta‐analysis consolidates that vitamin D‐deficient mothers are prone to developing gestational diabetes (Hu et al., 2018).

The literature published in the past two decades explaining the impact of maternal vitamin D level on mother and fetal health is reviewed. The most recent meta‐analysis has compiled almost 20 random clinical trials (RCTs) in which majority of evidences explicate that low vitamin D levels increase maternal insulin resistance and decrease infant birth weight. These variations in results are due to many confounding factors like ethnicity, study types, and criteria describing vitamin D threshold (Gallo et al., 2020). To further specify recommendations of vitamin D for healthy pregnancy and to verify the association, large‐scale, high‐quality, well‐designed, and randomized clinical trials are required.

### 4.2 Cesarean section

Vitamin D regulates body calcium level; thus, it also affects muscle contractions. Vitamin D receptors are situated in smooth muscles of uterine and skeletal muscles. Insufficient vitamin D can impair physical activity and muscle contractions, decreasing the strength of contractions and triggering obstructed or prolonged labor, which indicates the chances of C‐section (Hubeish et al., 2018). Additionally, it has also been manifested that vitamin D deficiency can cause pelvis malformation, leading toward C‐section (Gernand et al., 2015).

Few researches have been conducted to assess the correlation of vitamin D inadequacy with the risk of C‐section. A cohort study, including participants from different ethnic groups was directed by Loy et al. (2015), concluded that hypovitaminosis D affected the prevalence of C‐section in Indian and Chinese women but did not increase its chances in Malay women, thus overall indicating an inconsiderable interrelation. A. Gernand et al. (2017) also testified a null relation between suboptimal vitamin D levels and risk of C‐section. The survey of Gallo et al. (2020) further indicated that maternal vitamin D supplementation also does not interfere with the risk of C‐section.

According to recent research conducted by Hubeish et al. (2018), the ratio of C‐section by failure to descend, progress, and induction was threefolds more in vitamin D‐deficient (≤30 ng/ml) group as compared to control group. The study also postulated a substantial effect of vitamin D deficiency in postpartum hemorrhage and risk of uterine atony. This incongruity in findings can be attributed in terms of divergence in C‐section definition, either primary/secondary or elective/emergency indication (A. D. Gernand et al., 2015). It demands further RCTs to investigate the influence of vitamin D level on the rate of C‐section.

### 4.3 Pre‐eclampsia

Pre‐eclampsia (PE) is explained as hypertension (>140 mmHg/>90 mmHg) diagnosed between 20 and 34 weeks of gestation, along with proteinuria (urine protein ≥0.3 g/24 h twice measured ≥6 h apart) together with other complications like liver dysfunction, hematological disturbance, and renal and neurological problems (Mol et al., 2016). Pre‐eclampsia is explained pathogenically as a two‐stage disorder, the first being the production of angiogenic factors in maternal blood circulation, which leads to defective remodeling and trophoblastic invasion in spiral arteries, and subsequently to the abnormal implantation of the placenta. In the second stage, an abnormal balance between proangiogenic and antiangiogenic factors activates maternal inflammatory response, reducing vascularization and causing general endothelial dysfunction, affecting the functioning of all maternal organs (Gathiram & Moodley, 2016). Pre‐eclampsia thus increases the risk of fetal growth restriction and ultimately fetal death (Purswani et al., 2017).

The defective role of hypovitaminosis D in pre‐eclampsia can be explained through multiple biological processes including: Vitamin D acts as an immune modulator, moderating maternal immune response in the placenta and controlling the release of antiangiogenic factors in blood circulation, consequently causing elevation in blood pressure (Thorne & Fawzi, 2012; Woo et al., 2019). Vitamin D concentration is positively associated with serum calcium level, therefore insufficient vitamin D leads to low calcium levels, which subsequently stimulates parathyroid hormone and renin secretion, leading to vasoconstriction in vascular smooth muscle cells and endothelial cells, ultimately causing pre‐eclampsia; vasoconstrictive factors in the placenta are also released as a result of malfunctioning of effector and regulatory T cells, consequently causing hypertension and proteinuria (Hofmeyr et al., 2018).

Vitamin D has anti‐inflammatory characteristics which influence the inverse effect of calcium level on the occurrence of pre‐eclampsia (Purswani et al., 2017). Vitamin D3 also helps to avert cholesterol uptake by arterial smooth muscles and macrophages in the placenta of patient having pre‐eclampsia (Santorelli et al., 2019).

A case–cohort study was conducted to analyze the correlation of vitamin D concentration with pre‐eclampsia that demonstrated a direct effect of low 25(OH)D level and elevated risk of preeclampsia from mild to severe (Baca et al., 2016). A. Gernand et al. (2017), specifying the effects with respect to gestational weeks, deduced that women with low vitamin D levels had 2.4 times more chances of pre‐eclampsia onset on <35 weeks of gestation and there was no considerable relation during <37 weeks. According to Serrano et al. (2018), Fogacci et al. (2019), and Yuan et al. (2021), there is an evident lowering effect of optimal vitamin D on pregnancy‐induced hypertension.

On the contrary, several studies also claimed no significant relation between increasing intake of vitamin D and reduced risk for pre‐eclampsia. However, the evidence for helpful impact of vitamin D on pre‐eclampsia outweighs the negative results, and claims a comparatively casual relation (Dalmar et al., 2015; Pérez et al., 2015; Purswani et al., 2017).

### 4.4 Preterm delivery

Preterm delivery (PD) is defined as the early birth of a neonate before 37 weeks of pregnancy. Several genetic and immunological factors are considered as a causative factor (Yu et al., 2019). A broadly anticipated hypothesis links PD with inflammation and infections, including intra‐amniotic, maternal extrauterine, or intrauterine infections. Vitamin D owing to its anti‐inflammatory potential through nuclear factor kB inhibition, thereby decreases infections and PD risk (Monier et al., 2019).

A large number of studies attributed functions of VDR gene and associated placental signaling pathways with many maternal outcomes like growth and functioning. Patel et al. (2017) determined single nucleotide polymorphism (SNP) in VDR gene, and found that maternal vitamin D deficiency can elevate the risk of preterm delivery. In addition to PD, such SNPs can also affect fetal birth weight. In another study, Rosenfeld et al. (2017) investigated both maternal and fetal VDR polymorphism and found direct association with PD. Similarly, Javorski et al. (2018) studied two more polymorphisms and highlighted their effect in triggering spontaneous preterm birth.

A recent report by Kalok et al. (2020) consolidated that hypovitaminosis D increases the chances of impulsive preterm labor; and also raises the chances of cesarean section and preterm delivery. Maternal serum concentration of vitamin D ≥ 40 ng/ml is claimed to be linked with considerable reduction in PD risk in a large population of women.

Contrary results were also found in a recent retrospective cohort study, indicating that only vitamin D insufficiency cannot contribute to preterm birth (Yu et al., 2019). PD risk was found to be insignificant considering the concentration of vitamin D during 15th gestational week or in the first trimester (Boyle et al., 2016). Considering the evidences, a moderate impact of vitamin D in preterm delivery risk is suggested where the involvement of multiple confounders may also be suspected for which future studies should be designed for in‐depth analysis of the phenomenon.

### 4.5 Frequent miscarriages

Repeated pregnancy loss, a reproductive disorder, is defined as the demise of two or more pregnancies before 24 weeks of gestation, when the fetus has not yet reached a viable stage. It has been reported that the causes of 50% of the cases are unknown. Vitamin D concentration is regarded to have effects on the risk of miscarriages, in view of its immunomodulatory role (Gonçalves et al., 2018). Women with recurrent loss of pregnancy (RLP) were found to have low‐level VDR mRNA expression in serum, chorionic villi, and decidua during first trimester, endorsing the hypothesis (Yan et al., 2016). Genetic variation in maternal VDR FokI gene also consequently increases the risk of miscarriage (Hou et al., 2016).

Considering the part of vitamin D in regulating auto‐ and cellular immunity abnormalities, it is assumed that it may lower the chances of frequent pregnancy loss. Lower concentration of serum vitamin D in initial pregnancy signifies the risk of abortion (Jani et al., 2020). A cohort study, performed on women with spontaneous abortion rates, specified that low vitamin D has a potent effect in recurrent pregnancy loss. Results of pregnancy retention in more than half of the participating women indicated that vitamin D alone acted as an independent factor (Ji et al., 2019). A cross‐sectional study concluded that severe deficiency of vitamin D has damaging effects during early embryonic stages and can lead to loss of pregnancy (Wang et al., 2018). Thus, it is recommended to modulate vitamin D intake to avoid spontaneous pregnancy loss.

### 4.6 Postpartum depression

Postpartum depression (PPD), an acute mental condition, occurs after child delivery characterized by behavioral and emotional disturbance. There are several pathways that contribute to functions of vitamin D in depression pathogenesis. First, vitamin D plays a key role as a neurosteroid hormone regulating healthy nervous homeostasis and growth, providing protection and neuroplasticity (Berridge, 2017). Moreover, vitamin D also serves as an antioxidant by maintaining glutathione level in the brain. It influences the production of mood‐directing hormones, dopamine and norepinephrine, in the brain (Ali et al., 2018). In addition, it was also found that vitamin D regulates calcium ions in nerve cells that control the development of depressive symptoms. Thus, decreased vitamin D concentration increases neural calcium, leading to depression (Abedi et al., 2018). Furthermore, vitamin D receptors are located in the hippocampus and cingulate cortex of brain, which are involved in processing, memory storage, and planning (Wang et al., 2018). Endocrine Society recommends supplementation of vitamin D (2000 IU) during gestational and lactation period to meet elevated demands that may avoid the symptoms of PPD. Hence, vitamin D may play an important role in maintaining overall cognitive functions.

## 5 MATERNAL VITAMIN D DEFICIENCY AND ITS EFFECTS ON FETAL HEALTH

### 5.1 Reduced weight at birth

Underweight neonates (newborn child) are infants short for gestational age when they are small in size as compared to normal infants of the same age, specified by weight < 10th percentile for the equivalent gestational age. Low birth weight can subsequently lead to other comorbidities and disease outcomes in infancy and adult life including short stature, impaired neurocognitive development, poor academic performance, likelihood of cardiovascular disease (CVD), renal diseases, and diabetes (Chen et al., 2017). Vitamin D as a regulator of calcium balance and parathyroid hormone in the body can significantly impact fetal growth. Vitamin D can improve fetal outcomes in terms of birth weight, which is proved by some studies; meanwhile, some studies also gave opposite results (Ibrahim et al., 2019).

Scientific reports have found that increased concentration of vitamin D in maternal blood serum decreases the risk of short gestational age babies (SGA) and increases the normal birth weight bearing (Roth et al., 2017). According to Kalok et al. (2020), 25(OH)D concentration is directly correlated with baby weight at birth and gestational age. The risk of SGA is not influenced by the concentration of vitamin D at 15th week of gestation (Boyle et al., 2016). Moreover, any polymorphism in VDR gene produces outcomes related to birth weight and gestational age (Pereira et al., 2019). According to Kim et al. (2019), 78.6% of the preterm babies included in their study were vitamin D deficient.

Most of the evidences suggest a direct relation of insufficient vitamin D on prevailing risk of low birth weight and SGA babies. However, there is still need for adequate randomized clinical trials to determine the underlying pathogenesis and the effect of calcitriol supplementation on fetal outcomes.

### 5.2 Asthma, COVID, and other respiratory infections

Asthma is an inflammatory disease associated with airway hyperresponsiveness followed by symptoms like dyspnea, cough, wheezing, and chest congestion (Jensen et al., 2019). Obesity, environmental pollutants, allergens, tobacco use, infections, and many other factors like elevated IgE antibodies, mast cell deregulation, hypereosinophilia, imbalance of immunological factors, and growth factors may contribute to pathogenesis of asthma (Jartti et al., 2020).

A study revealed a remarkable change in proinflammatory cytokines (IL‐6 & 8, IFN‐γ, and IL‐1β) in response to stimuli, and an increased protein expression of genes TLR2, TLR9, and IL‐17 after acquired immunity stimulation, when mothers were supplemented with 4400 IU calcitriol, thus giving aid in dealing with asthma and related infections (Hornsby et al., 2018). A possible explanation for this effect is that vitamin D has a potential effect on fetal pulmodevelopment by regulating mesenchymal and epithelial cells of alveoli. Secondly, VDR are also expressed in the type 2 alveolar cells, which are responsible for surfactant mucus secretion responding to vitamin D stimuli (Kim et al., 2019).

Most of the respiratory tract infections are viral, e.g., influenza, coronavirus, and respiratory syncytial virus (RSV), where infections in lower tract are usually preceded by upper tract infections (Jartti et al., 2020). Vitamin D, owing to its immunological response as an antiviral candidate, helps in reducing morbidity rate of respiratory infections among the population (Morris et al., 2016). It is predicted to be beneficial for mothers and newborns during COVID‐19 pandemic to boost the body immunity against infectious diseases (Mirzadeh & Khedmat, 2020).

In an animal trial, it was suggested that maternal vitamin D deficiency can lead to malformation of lungs, hyperreactivity of airways, and impaired lung mechanics in infancy, inferring that disrupted prenatal vitamin D signaling elevates respiratory dysfunction in late childhood (Mandell et al., 2020). Kim et al. (2019) also indicated the association of vitamin D deficiency at birth with respiratory infections, but could not explain the mechanism. A meta‐analysis compiling 10 observational studies, including 23,030 mother–child pairs, suggested that an increase in vitamin D intake during gestational period can have a defensive role for asthma and wheezing in children (Shi et al., 2021).

However, a follow‐up comparative study of 6 years' trial determined asthma risk in children born to vitamin D sufficient and insufficient mothers, and found that vitamin D solely cannot control these respiratory complications (Yadama et al., 2020). Taking into account all these evidences, it can be suggested that optimal vitamin D requirements should be fulfilled to potentially prevent respiratory tract infections.

### 5.3 Physical measurements and bone growth

An optimal supply of energy and essential nutrients is necessary for appropriate intrauterine fetal growth and development. The role of vitamin D in maintaining calcium concentration in the body is considered essential for regularized musculoskeletal growth; it is hypothesized that concentration of maternal vitamin D level can control neonates' bone development, length, and density. A prospective cohort study conducted by Sarma et al. (2018) concluded that healthy skeletal development of fetus is significantly altered by vitamin D insufficiency. An RCT with a double‐blinding technique proved a positive impact of high‐dose maternal vitamin D supplementation on ameliorated mineralization of bones as compared to standardized dosage (Brustad et al., 2020). Brustad et al. (2020) also reported an improved head bone mineral content and density, tracked up to 6 years, but vitamin D supplementation did not affect the head circumference in his study. Length of femur bone and infant length at birth are also affected and these lengths might be shorter in neonates born to vitamin D‐deficient mothers, thus depicting a significant relation (Sarma et al., 2018).

Laird et al. (2017) investigated maternal vitamin D concentration and its effect on neonate anthropometrics (head circumference and birth weight) and neurocognitive developmental consequences in children of about 5 years of age. For the purpose, serum 25(OH)D concentration was determined from blood samples at the time of delivery. Average gestational period was 39 weeks and none of the mother was vitamin D deficient. Various linear regression models weighed the association between maternal vitamin D concentration, neonate anthropometrics and neurocognitive development. The results showed insignificant effect of maternal vitamin D on children’s neurological and anthropometric development i.e., higher concentration of vitamin D do not have a preventive role.

Neonates vitamin D status at birth can actuate anthropometric indices and can also result in diabetes type 2 and obesity (Keller et al., 2018). Contrasting results were also found as shown in a cohort study of gravid women from diverse ethnic groups with hypovitaminosis D. It presented an insignificant influence on infant's physical measurements (Eggemoen et al., 2017). A study conducted at the genetic level by using SNPs claimed no significant interrelation between vitamin D‐associated genes polymorphism and neonates anthropometric indices, except for head circumference (HC), where vitamin D‐deficient mothers (carrying more altered genes) gave birth to babies with HC < 35 cm (Aji et al., 2020). Contrary to these findings, Casey et al. (2018) deduced that high maternal and neonatal vitamin D can cause detrimental outcomes in growth. Infants’ mothers with vitamin D > 125 nmol/L were thin, shorter, light in weight, and had smaller head circumference as compared to normal, at 6 and at 12 months of age. Overall, it may be said that there are inconsistent rulings for maternal vitamin D status that may affect neonatal anthropometrics, hence further verification through stringent trials is required.

### 5.4 Autism and neurodevelopment

Autism spectrum disorder is a heterogeneous disease, with an enlarged brain due to abnormal growth and differentiation of nerve cells, causing symptoms of impaired communication, hyperactivity in response to sensory stimuli, and repeated movements (Principi & Esposito, 2020). It also involves altered cytokine concentration in serum, elevated by oxidative stress of disease. Vitamin D, an immunomodulator, augments dendritic lymphocytes and anti‐inflammatory cytokine proteins (IL‐10); its neurodefensive activity is provided by increasing the release of neurotropins (Cannell, 2017; Lisi et al., 2020). In addition, vitamin D can modulate cell mitosis via cyclin‐dependent kinase inhibitors and cyclin D1 (Ali et al., 2018). Calcitriol (supplemental vitamin D3) exacerbates genetic repair process, increases glutathione (a chief antioxidant), shields mitochondria of neurons, and upregulates T‐regulatory cells. Autism symptoms are also modulated by vitamin D through genetic mediation of enzymes by limiting the rate of serotonin release, increasing central tryptophan hydroxylase TPH2, and decreasing peripheral TPH1 (Cannell, 2017).

Vitamin D association with brain development can be predicted by the findings that enzymes metabolizing vitamin D, CYP27B1 and CYP24A1, are localized in brain cells like the cortex region and Purkinje cells, indicating the independent role of vitamin D in brain. Moreover, VDR and an important binding receptor protein, i.e., 1,25D3‐MARRS, are detected in fetal and adult brain (Ali & Nanji, 2017). Further studies have highlighted an eminent role of vitamin D in nerve cell differentiation and apoptosis, dopamine production, axonal integration, immunological regulation, and genetic transcription (Lisi et al., 2020).

Recently, extensive scientific literature has been published determining the role of vitamin D in brain development. According to Vinkhuyzen et al. (2018) and Lee et al. (2021), maternal serum concentration of vitamin D has effects on neurological development, and its deficiency can increase the risk of neurological disorders.

A meta‐analysis including 25 studies indicated the influence of maternal and fetal vitamin D on neurodevelopment supporting significant evidence in favor of vitamin D's role in autism‐associated symptoms and attention deficit and hyperactivity disorder (ADHD). Sucksdorff et al. (2021) also demonstrated an increased risk of attention deficit and hyperactivity disorder (ADHD) in infants born to vitamin D‐deficient mothers. Ability to perceive movements and responsiveness was not significantly affected by mother's circulating 25(OH)D, but the study showed noteworthy outcomes on infants' attentiveness and executive operation (Brouwer‐Brolsma et al., 2018). There are also a few researches supporting its defensive role in language and behavior developing skills (Lisi et al., 2020). According to López‐Vicente et al. (2019), an insignificant association was found among children of 5–18 age group, when maternal serum concentration of calcitriol was measured and its effects on autism symptoms, behavioral responses, and ADHD were determined. In a most recent systematic review including 32 studies, 12 mother–infant observational studies showed positive association while 15 showed no correlation of maternal vitamin D on neonates' neurological development and behavioral responses (Mutua et al., 2020).

### 5.5 Adipogenesis

Poor maternal diet can lead to obesity and metabolic problems in neonates, triggered by hormonal imbalance during catch‐up growth and long‐term mild inflammation of white adipose tissues (WAT). Obesity, excessive adipogenesis, is a disorder that arises either by the development of new fat cells (adipocytes) or the increase in size/accumulation of fat in already present adipocytes, accompanied by the release of signaling inflammatory proteins, cytokines (Mehmood & Papandreou, 2016). The increase in the number of fat cells largely occurs during the fetal growth and early infancy period, leading to adverse health outcomes of obesity (Santamaria et al., 2018).Vitamin D and its receptor (VDR) have been found to influence adipocyte procreation, as VDR are present abundantly on adipocyte cells (Wen et al., 2018). Underlying pathways and potential role of vitamin D‐mediated signaling are still uncertain, demanding animal and human trials to testify the interrelation. Various findings showed both positive and negative effects of vitamin D on adipogenesis (Vickers, 2014).

Neonatal vitamin D concentration also contributes toward the adipocyte cell development of infant body and has inverse relationship: greater the concentration, lower will be the adipogenesis (Horan et al., 2017). At genetic level, maternal vitamin D insufficiency is found to foster genetic mutation in nonobese neonates, and can alter the genes associated with adipose tissue development (Belenchia et al., 2018). Moreover, the deficiency of this vitamin may improve differential augmentation of preadipocytes, which can induce methylation in genes, causing obesity in offspring (Wen et al., 2018). According to an animal study by Schutkowski et al. (2018), vitamin D has not advocated a significant impact in fetal adipogenesis. A cohort study conducted by Larsen et al. (2020) found an opposing result tracked up to 3 years of child birth, but unconvincing relation between maternal or cord blood vitamin D and obesity in children was found.

Wen et al. (2018) conducted an animal study that identified the elevated preadipocytes production and differentiation in vitamin D‐deficient rats, which might be due to methylation of genes, and in the end leading toward obesity. Most recently, an animal study was conducted to determine the relation of maternal hypovitaminosis D with obesity and significant changes were observed in vitamin D‐deficient mice. It led to an increase in adipocytes, altered fat and carbohydrate metabolism, elevated pro‐inflammatory cytokines, and decreased anti‐inflammatory cytokines along with decreased level of IL‐10, IL‐4, and raised INF‐β/ TNF‐α levels, hence, revealing the influence of vitamin D deficiency on obesity development (Li et al., 2021).

Further investigations and human clinical trials are still required to establish the firm grounds for understanding underlying mechanisms that may assist in developing strategies to combat maternal and fetal health implications.

## 6 CONCLUSIONS

Hypovitaminosis D has become a general public health problem, especially for women of reproductive age, children, and older adults as they are more vulnerable. Vitamin D deficiency is also likely to be prevalent due to different sun exposure, altitude, and ethnicity, so greater attention should be given to meet the bodily requirement of vitamin D. It has a potential importance, evident from many researches, in maintaining mother–fetal health during antenatal and postnatal period, limiting detrimental outcomes. Vitamin D has foreground immunomodulatory, skeletal and calcium regulatory, and anti‐inflammatory effect on the body due to which not only can it expedite the prognosis of gestational diabetes, pre‐eclampsia, recurrent abortion rate, and postpartum depression in the mother but it can also clearly direct the respiratory tract infections, fetal weight at birth, adipogenesis, and neurological development in neonates. It can be postulated that vitamin D‐rich foods and supplementation can control the symptoms of coronavirus infection in general population, including gravid mothers and infants. Further studies should be conducted in this facet. The underlying mechanisms are not well understood, and still more clear insights into vitamin D etiology are to be discovered. Scientific field should welcome further researches, clinical trials with placebo double‐blinding, genetic analysis, and more detailed investigations to scrutinize hidden mechanisms for devising strategies to overcome maternal and fetal health implications.

## ACKNOWLEDGEMENT

None.

## Data Availability

The dataset supporting the conclusions of this article is included within the article.

## References

[fsn32948-bib-0001] Abedi, P. , Bovayri, M. , Fakhri, A. , & Jahanfar, S. (2018). The relationship between vitamin D and postpartum depression in reproductive‐aged Iranian women. Journal of Medicine and Life, 11(4), 286–292. 30894884 10.25122/jml-2018-0038 PMC6418338

[fsn32948-bib-0002] Aji, A. S. , Erwinda, E. , Rasyid, R. , Yusrawati, Y. , Malik, S. G. , Alathari, B. , Lovegrove, J. A. , Lipoeto, N. I. , & Vimaleswaran, K. S. (2020). A genetic approach to study the relationship between maternal vitamin D status and newborn anthropometry measurements: The vitamin D pregnant mother (VDPM) cohort study. Journal of Diabetes & Metabolic Disorders, 19(1), 91–103. 32548071 10.1007/s40200-019-00480-5 PMC7270445

[fsn32948-bib-0003] Ali, A. , Cui, X. , & Eyles, D. (2018). Developmental vitamin D deficiency and autism: Putative pathogenic mechanisms. The Journal of Steroid Biochemistry and Molecular Biology, 175, 108–118. 28027915 10.1016/j.jsbmb.2016.12.018

[fsn32948-bib-0004] Ali, N. S. , & Nanji, K. (2017). A review on the role of vitamin D in asthma. Cureus, 9(5), e1288. 10.7759/cureus.1288 28680776 PMC5491340

[fsn32948-bib-0005] Anwar, H. , Rasul, A. , Iqbal, J. , Ahmad, N. , Imran, A. , Malik, S. A. , Ijaz, F. , Akram, R. , Maqbool, J. , Sajid, F. , Sun, T. , Hussain, G. , & Manzoor, M. F. (2021). Dietary biomolecules as promising regenerative agents for peripheral nerve injury: An emerging nutraceutical‐based therapeutic approach. Journal of Food Biochemistry, 45(12), e13989. 34719796 10.1111/jfbc.13989

[fsn32948-bib-0006] Arshad, R. , Gulshad, L. , Haq, I. U. , Farooq, M. A. , al‐Farga, A. , Siddique, R. , Manzoor, M. F. , & Karrar, E. (2021). Nanotechnology: A novel tool to enhance the bioavailability of micronutrients. Food science nutrition, 9(6), 3354–3361. 34136200 10.1002/fsn3.2311 PMC8194941

[fsn32948-bib-0007] Ateş, S. , Aydın, S. , Karasu, A. F. G. , & Dane, B. (2017). Association between maternal vitamin D status and risk of gestational diabetes mellitus in pregnant women. Haseki Tip Bulteni, 55(1), 15–20.

[fsn32948-bib-0008] Baca, K. M. , Simhan, H. N. , Platt, R. W. , & Bodnar, L. M. (2016). Low maternal 25‐hydroxyvitamin D concentration increases the risk of severe and mild preeclampsia. Annals of Epidemiology, 26(12), 853–857. e851. 27818017 10.1016/j.annepidem.2016.09.015 PMC7848792

[fsn32948-bib-0009] Belenchia, A. M. , Jones, K. L. , Will, M. , Beversdorf, D. Q. , Vieira‐Potter, V. , Rosenfeld, C. S. , & Peterson, C. A. (2018). Maternal vitamin D deficiency during pregnancy affects expression of adipogenic‐regulating genes peroxisome proliferator‐activated receptor gamma (PPARγ) and vitamin D receptor (VDR) in lean male mice offspring. European Journal of Nutrition, 57(2), 723–730. 28004271 10.1007/s00394-016-1359-x PMC6643277

[fsn32948-bib-0010] Berridge, M. J. (2017). Vitamin D and depression: Cellular and regulatory mechanisms. Pharmacological Reviews, 69(2), 80–92. 28202503 10.1124/pr.116.013227

[fsn32948-bib-0011] Boyle, V. T. , Thorstensen, E. B. , Mourath, D. , Jones, M. B. , McCowan, L. M. , Kenny, L. C. , & Baker, P. N. (2016). The relationship between 25‐hydroxyvitamin D concentration in early pregnancy and pregnancy outcomes in a large, prospective cohort. British Journal of Nutrition, 116(8), 1409–1415. 27753425 10.1017/S0007114516003202

[fsn32948-bib-0012] Brouwer‐Brolsma, E. M. , Vrijkotte, T. G. , & Feskens, E. J. (2018). Maternal vitamin D concentrations are associated with faster childhood reaction time and response speed, but not with motor fluency and flexibility, at the age of 5–6 years: The Amsterdam born children and their development (ABCD) study. British Journal of Nutrition, 120(3), 345–352. 29843832 10.1017/S0007114518001319

[fsn32948-bib-0013] Brustad, N. , Garland, J. , Thorsen, J. , Sevelsted, A. , Krakauer, M. , Vinding, R. K. , Stokholm, J. , Bønnelykke, K. , Bisgaard, H. , & Chawes, B. L. (2020). Effect of high‐dose vs standard‐dose vitamin D supplementation in pregnancy on bone mineralization in offspring until age 6 years: A prespecified secondary analysis of a double‐blinded, randomized clinical trial. JAMA Pediatrics, 174(5), 419–427. 32091548 10.1001/jamapediatrics.2019.6083 PMC7042912

[fsn32948-bib-0014] Cannell, J. J. (2017). Vitamin D and autism, what's new? Reviews in Endocrine and Metabolic Disorders, 18(2), 183–193. 28217829 10.1007/s11154-017-9409-0

[fsn32948-bib-0015] Casey, C. , McGinty, A. , Holmes, V. A. , Patterson, C. C. , Young, I. S. , & McCance, D. R. (2018). Maternal vitamin D and neonatal anthropometrics and markers of neonatal glycaemia: Belfast hyperglycemia and adverse pregnancy outcome (HAPO) study. British Journal of Nutrition, 120(1), 74–80. 29936925 10.1017/S0007114518001320 PMC6023417

[fsn32948-bib-0016] Cashman, K. D. , van den Heuvel, E. G. , Schoemaker, R. J. , Prévéraud, D. P. , Macdonald, H. M. , & Arcot, J. (2017). 25‐Hydroxyvitamin D as a biomarker of vitamin D status and its modeling to inform strategies for prevention of vitamin D deficiency within the population. Advances in Nutrition, 8(6), 947–957. 29141976 10.3945/an.117.015578 PMC5682995

[fsn32948-bib-0017] Chen, Y. , Zhu, B. , Wu, X. , Li, S. , & Tao, F. (2017). Association between maternal vitamin D deficiency and small for gestational age: Evidence from a meta‐analysis of prospective cohort studies. BMJ Open, 7(8), e016404. 10.1136/bmjopen-2017-016404 PMC5629738 28844987

[fsn32948-bib-0018] Curtis, E. M. , Moon, R. J. , Harvey, N. C. , & Cooper, C. (2018). Maternal vitamin D supplementation during pregnancy. British Medical Bulletin, 126(1), 57–77. 29684104 10.1093/bmb/ldy010 PMC6003599

[fsn32948-bib-0019] Dalmar, A. , Raff, H. , Chauhan, S. P. , Singh, M. , & Siddiqui, D. S. (2015). Serum 25‐hydroxyvitamin D, calcium, and calcium‐regulating hormones in preeclamptics and controls during first day postpartum. Endocrine, 48(1), 287–292. 24853885 10.1007/s12020-014-0296-9

[fsn32948-bib-0020] Eggemoen, Å. R. , Jenum, A. K. , Mdala, I. , Knutsen, K. V. , Lagerløv, P. , & Sletner, L. (2017). Vitamin D levels during pregnancy and associations with birth weight and body composition of the newborn: A longitudinal multiethnic population‐based study. British Journal of Nutrition, 117(7), 985–993. 28468694 10.1017/S000711451700068X

[fsn32948-bib-0021] Flood‐Nichols, S. K. , Tinnemore, D. , Huang, R. R. , Napolitano, P. G. , & Ippolito, D. L. (2015). Vitamin D deficiency in early pregnancy. PLoS One, 10(4), e0123763. 25898021 10.1371/journal.pone.0123763 PMC4405493

[fsn32948-bib-0022] Fogacci, S. , Fogacci, F. , & Cicero, A. (2019). Does vitamin d supplementation reduce the risk of pre‐eclampsia? Atherosclerosis, 287, e88.

[fsn32948-bib-0023] Gallo, S. , McDermid, J. M. , Al‐Nimr, R. I. , Hakeem, R. , Moreschi, J. M. , Pari‐Keener, M. , Stahnke, B. , Papoutsakis, C. , Handu, D. , & Cheng, F. W. (2020). Vitamin D supplementation during pregnancy: An evidence analysis center systematic review and meta‐analysis. Journal of the Academy of Nutrition and Dietetics, 120(5), 898–924. e894. 31669079 10.1016/j.jand.2019.07.002

[fsn32948-bib-0024] Gathiram, P. , & Moodley, J. (2016). Pre‐eclampsia: Its pathogenesis and pathophysiolgy: Review articles. Cardiovascular Journal of Africa, 27(2), 71–78. 27213853 10.5830/CVJA-2016-009 PMC4928171

[fsn32948-bib-0025] Gernand, A. , Simhan, H. , Baca, K. , Caritis, S. , & Bodnar, L. (2017). Vitamin D, pre‐eclampsia, and preterm birth among pregnancies at high risk for pre‐eclampsia: An analysis of data from a low‐dose aspirin trial. BJOG: An International Journal of Obstetrics & Gynaecology, 124(12), 1874–1882. 27704679 10.1111/1471-0528.14372 PMC7845451

[fsn32948-bib-0026] Gernand, A. D. , Klebanoff, M. A. , Simhan, H. N. , & Bodnar, L. M. (2015). Maternal vitamin D status, prolonged labor, cesarean delivery and instrumental delivery in an era with a low cesarean rate. Journal of Perinatology, 35(1), 23–28. 25102320 10.1038/jp.2014.139 PMC4281279

[fsn32948-bib-0027] Gonçalves, D. R. , Braga, A. , Braga, J. , & Marinho, A. (2018). Recurrent pregnancy loss and vitamin D: A review of the literature. American Journal of Reproductive Immunology, 80(5), e13022. 30051540 10.1111/aji.13022

[fsn32948-bib-0028] Haq, I. U. , Rajoka, M. S. R. , Asim Shabbir, M. , Umair, M. , llah, I. U. , Manzoor, M. F. , Nemat, A. , Abid, M. , Khan, M. R. , & Aadil, R. M. (2021). Role of stilbenes against insulin resistance: A review. Food Science Nutrition, 9(11), 6389–6405. 34760269 10.1002/fsn3.2553 PMC8565239

[fsn32948-bib-0029] Hofmeyr, G. J. , Lawrie, T. A. , Atallah, Á. N. , & Torloni, M. R. (2018). Calcium supplementation during pregnancy for preventing hypertensive disorders and related problems. Cochrane Database of Systematic Reviews, 10(10), Cd001059. 10.1002/14651858.CD001059.pub5 30277579 PMC6517256

[fsn32948-bib-0030] Horan, M. K. , Donnelly, J. M. , McKenna, M. J. , Crosbie, B. , Kilbane, M. T. , & McAuliffe, F. M. (2017). An examination of whether associations exist between maternal and neonatal 25OHD and infant size and adiposity at birth, 6–9 months and 2–2.5 years of age–a longitudinal observational study from the ROLO study. BMC nutrition, 3(1), 1–10. 10.1186/s40795-017-0184-9 PMC7050699 32153842

[fsn32948-bib-0031] Hornsby, E. , Pfeffer, P. E. , Laranjo, N. , Cruikshank, W. , Tuzova, M. , Litonjua, A. A. , Weiss, S. T. , Carey, V. J. , O'Connor, G. , & Hawrylowicz, C. (2018). Vitamin D supplementation during pregnancy: Effect on the neonatal immune system in a randomized controlled trial. Journal of Allergy and Clinical Immunology, 141(1), 269–278. e261. 28552588 10.1016/j.jaci.2017.02.039

[fsn32948-bib-0032] Hou, W. , Yan, X. , Bai, C. , Zhang, X. , Hui, L. , & Yu, X. (2016). Decreased serum vitamin D levels in early spontaneous pregnancy loss. European Journal of Clinical Nutrition, 70(9), 1004–1008. 27222154 10.1038/ejcn.2016.83 PMC5023787

[fsn32948-bib-0033] Hu, L. , Zhang, Y. , Wang, X. , You, L. , Xu, P. , Cui, X. , Zhu, L. , Ji, C. , Guo, X. , & Wen, J. (2018). Maternal vitamin D status and risk of gestational diabetes: A meta‐analysis. Cellular Physiology and Biochemistry, 45(1), 291–300. 29402818 10.1159/000486810

[fsn32948-bib-0034] Hubeish, M. , Al Husari, H. , Itani, S. E. , El Tal, R. , Tamim, H. , & Abou Saleh, S. (2018). Maternal vitamin D level and rate of primary cesarean section. Journal of Clinical Gynecology and Obstetrics, 7(2), 43–51.

[fsn32948-bib-0035] Ibrahim, M. , Abd Elrahman, R. M. , & El‐Kateb, M. (2019). The association between gestational vitamin D deficiency and preterm birth: A case control study. Evidence Based Women's Health Journal, 9(4), 605–613.

[fsn32948-bib-0036] Jani, R. , Knight‐Agarwal, C. R. , Bloom, M. , & Takito, M. Y. (2020). The association between pre‐pregnancy body mass index, perinatal depression and maternal vitamin D status: Findings from an Australian cohort study. International Journal of Women's Health, 12, 213–219. 10.2147/IJWH.S239267 PMC7105885 32273777

[fsn32948-bib-0037] Jartti, T. , Bønnelykke, K. , Elenius, V. , & Feleszko, W. (2020). Role of viruses in asthma. Seminars in Immunopathology, 42(1), 61–74. 10.1007/s00281-020-00781-5 31989228 PMC7066101

[fsn32948-bib-0038] Javorski, N. , Lima, C. , Silva, L. , Crovella, S. , & de Azêvedo Silva, J. (2018). Vitamin D receptor (VDR) polymorphisms are associated to spontaneous preterm birth and maternal aspects. Gene, 642, 58–63. 29128634 10.1016/j.gene.2017.10.087

[fsn32948-bib-0039] Jensen, M. E. , Murphy, V. , Gibson, P. , Mattes, J. , & Camargo, C., Jr. (2019). Vitamin D status in pregnant women with asthma and its association with adverse respiratory outcomes during infancy. The Journal of Maternal‐Fetal & Neonatal Medicine, 32(11), 1820–1825. 29303025 10.1080/14767058.2017.1419176

[fsn32948-bib-0040] Ji, J. , Zhai, H. , Zhou, H. , Song, S. , Mor, G. , & Liao, A. (2019). The role and mechanism of vitamin D‐mediated regulation of Treg/Th17 balance in recurrent pregnancy loss. American Journal of Reproductive Immunology, 81(6), e13112. 30903715 10.1111/aji.13112

[fsn32948-bib-0041] Kalok, A. , Aziz, N. , Malik, D. , Shah, S. , Nasuruddin, D. , Omar, M. , Ismail, N. A. M. , & Shafiee, M. (2020). Maternal serum vitamin D and spontaneous preterm birth. Clinical and Experimental Obstetrics & Gynecology, 47, 16–20.

[fsn32948-bib-0042] Keller, A. , Händel, M. N. , Frederiksen, P. , Jacobsen, R. , Cohen, A. S. , McGrath, J. J. , & Heitmann, B. L. (2018). Concentration of 25‐hydroxyvitamin D from neonatal dried blood spots and the relation to gestational age, birth weight and Ponderal index: The D‐tect study. British Journal of Nutrition, 119(12), 1416–1423. 29690937 10.1017/S0007114518000879

[fsn32948-bib-0043] Kim, I. , Kim, S. S. , Song, J. I. , Yoon, S. H. , Park, G. Y. , & Lee, Y.‐W. (2019). Association between vitamin D level at birth and respiratory morbidities in very‐low‐birth‐weight infants. Korean Journal of Pediatrics, 62(5), 166–172. 30360037 10.3345/kjp.2018.06632 PMC6528057

[fsn32948-bib-0044] Lacroix, M. , Battista, M.‐C. , Doyon, M. , Houde, G. , Ménard, J. , Ardilouze, J.‐L. , Hivert, M. F. , & Perron, P. (2014). Lower vitamin D levels at first trimester are associated with higher risk of developing gestational diabetes mellitus. Acta Diabetologica, 51(4), 609–616. 24526261 10.1007/s00592-014-0564-4

[fsn32948-bib-0045] Laird, E. , Thurston, S. W. , Van Wijngaarden, E. , Shamlaye, C. F. , Myers, G. J. , Davidson, P. W. , Watson, G. E. , McSorley, E. M. , Mulhern, M. S. , Yeates, A. J. , Ward, M. , McNulty, H. , & Yeates, A. J. (2017). Maternal vitamin D status and the relationship with neonatal anthropometric and childhood neurodevelopmental outcomes: Results from the Seychelles child development nutrition study. Nutrients, 9(11), 1235. 10.3390/nu9111235 PMC5707707 29137132

[fsn32948-bib-0046] Larqué, E. , Morales, E. , Leis, R. , & Blanco‐Carnero, J. E. (2018). Maternal and foetal health implications of vitamin D status during pregnancy. Annals of Nutrition and Metabolism, 72(3), 179–192. 29533937 10.1159/000487370

[fsn32948-bib-0047] Larsen, S. D. , Christensen, M. E. , Dalgård, C. , Lykkedegn, S. , Andersen, L. B. , Andersen, M. S. , Glintborg, D. , & Christesen, H. T. (2020). Pregnancy or cord 25‐hydroxyvitamin D is not associated with measures of body fat or adiposity in children from three months to three years of age. An Odense child cohort study. Clinical Nutrition, 39(6), 1832–1839. 31471164 10.1016/j.clnu.2019.07.023

[fsn32948-bib-0048] Lee, B. K. , Eyles, D. W. , Magnusson, C. , Newschaffer, C. J. , McGrath, J. J. , Kvaskoff, D. , & Gardner, R. M. (2021). Developmental vitamin D and autism spectrum disorders: Findings from the Stockholm youth cohort. Molecular Psychiatry, 26(5), 1578–1588. 31695167 10.1038/s41380-019-0578-y PMC7200274

[fsn32948-bib-0049] Li, P. , Li, P. , Liu, Y. , Liu, W. , Zha, L. , Chen, X. , Zheng, R. , Qi, K. , & Zhang, Y. (2021). Maternal vitamin D deficiency increases the risk of obesity in male offspring mice by affecting the immune response. Nutrition, 87‐88, 111191. 10.1016/j.nut.2021.111191 33744641

[fsn32948-bib-0050] Lisi, G. , Ribolsi, M. , Siracusano, A. , & Niolu, C. (2020). Maternal vitamin D and its role in determining fetal origins of mental health. Current Pharmaceutical Design, 26(21), 2497–2509. 32370709 10.2174/1381612826666200506093858

[fsn32948-bib-0051] Liu, N. , & Hewison, M. (2012). Vitamin D, the placenta and pregnancy. Archives of Biochemistry and Biophysics, 523(1), 37–47. 22155151 10.1016/j.abb.2011.11.018

[fsn32948-bib-0052] López‐Vicente, M. , Sunyer, J. , Lertxundi, N. , González, L. , Rodríguez‐Dehli, C. , Espada Sáenz‐Torre, M. , Vrijheid, M. , Tardón, A. , Llop, S. , Torrent, M. , Ibarluzea, J. , & Guxens, M. (2019). Maternal circulating vitamin D(3) levels during pregnancy and behaviour across childhood. Scientific Reports, 9(1), 14792. 10.1038/s41598-019-51325-3 31616023 PMC6794315

[fsn32948-bib-0053] Loy, S. L. , Lek, N. , Yap, F. , Soh, S. E. , Padmapriya, N. , Tan, K. H. , Biswas, A. , Yeo, G. S. , Kwek, K. , Gluckman, P. D. , Godfrey, K. M. , Saw, S. M. , Müller‐Riemenschneider, F. , Chong, Y. S. , Chong, M. F. , Chan, J. K. , & Growing Up in Singapore Towards Healthy Outcomes (GUSTO) study group . (2015). Association of maternal vitamin D status with glucose tolerance and caesarean section in a multi‐ethnic Asian cohort: The growing up in Singapore towards healthy outcomes study. PLoS One, 10(11), e0142239. 26571128 10.1371/journal.pone.0142239 PMC4646602

[fsn32948-bib-0054] Mandell, E. W. , Ryan, S. , Seedorf, G. J. , Gonzalez, T. , Smith, B. J. , Fleet, J. C. , & Abman, S. H. (2020). Maternal vitamin D deficiency causes sustained impairment of lung structure and function and increases susceptibility to hyperoxia‐induced lung injury in infant rats. American Journal of Respiratory Cell and Molecular Biology, 63(1), 79–91. 32135073 10.1165/rcmb.2019-0295OC PMC7328245

[fsn32948-bib-0055] Manzoor, M. F. , Hussain, A. , Sameen, A. , Sahar, A. , Khan, S. , Siddique, R. , Aadil, R. M. , & Xu, B. (2021). Novel extraction, rapid assessment and bioavailability improvement of quercetin: A review. Ultrasonics Sonochemistry, 78, 105686. 34358980 10.1016/j.ultsonch.2021.105686 PMC8350193

[fsn32948-bib-0056] Mehmood, Z. H. , & Papandreou, D. (2016). An updated mini review of vitamin D and obesity: Adipogenesis and inflammation state. Open Access Maced J Med Sci, 4(3), 526–532. 10.3889/oamjms.2016.103 27703587 PMC5042647

[fsn32948-bib-0057] Mirzadeh, M. , & Khedmat, L. (2020). Pregnant women in the exposure to COVID‐19 infection outbreak: The unseen risk factors and preventive healthcare patterns. The Journal of Maternal‐Fetal & Neonatal Medicine, 35(7), 1377–1378. 10.1080/14767058.2020.1749257 32223477

[fsn32948-bib-0058] Mol, B. W. , Roberts, C. T. , Thangaratinam, S. , Magee, L. A. , De Groot, C. J. , & Hofmeyr, G. J. (2016). Pre‐eclampsia. The Lancet, 387(10022), 999–1011. 10.1016/S0140-6736(15)00070-7 26342729

[fsn32948-bib-0059] Monier, I. , Baptiste, A. , Tsatsaris, V. , Senat, M.‐V. , Jani, J. , Jouannic, J.‐M. , Winer, N. , Elie, C. , Souberbielle, J. C. , Zeitlin, J. , & Zeitlin, J. (2019). First trimester maternal vitamin D status and risks of preterm birth and small‐for‐gestational age. Nutrients, 11(12), 3042. 10.3390/nu11123042 PMC6950733 31847068

[fsn32948-bib-0060] Morris, S. K. , Pell, L. G. , Rahman, M. Z. , Dimitris, M. C. , Mahmud, A. , Islam, M. M. , Ahmed, T. , Pullenayegum, E. , Kashem, T. , Shanta, S. S. , Gubbay, J. , Papp, E. , Science, M. , Zlotkin, S. , & Shanta, S. S. (2016). Maternal vitamin D supplementation during pregnancy and lactation to prevent acute respiratory infections in infancy in Dhaka, Bangladesh (MDARI trial): Protocol for a prospective cohort study nested within a randomized controlled trial. BMC Pregnancy and Childbirth, 16(1), 1–10. 27737646 10.1186/s12884-016-1103-9 PMC5064894

[fsn32948-bib-0061] Mutua, A. M. , Mogire, R. M. , Elliott, A. M. , Williams, T. N. , Webb, E. L. , Abubakar, A. , & Atkinson, S. H. (2020). Effects of vitamin D deficiency on neurobehavioural outcomes in children: A systematic review. Wellcome Open Research, 5, 28. 10.12688/wellcomeopenres.15730.1 32399499 PMC7194460

[fsn32948-bib-0062] Palacios, C. , De‐Regil, L. M. , Lombardo, L. K. , & Peña‐Rosas, J. P. (2016). Vitamin D supplementation during pregnancy: Updated meta‐analysis on maternal outcomes. The Journal of Steroid Biochemistry and Molecular Biology, 164, 148–155. 26877200 10.1016/j.jsbmb.2016.02.008 PMC5357731

[fsn32948-bib-0063] Patel, H. V. , Patel, N. H. , & Sodagar, N. R. (2017). Vitamin d receptor (VDR) gene polymorphism and maternal vitamin d deficiency in indian women with preterm birth (PTB). Asian Journal of Pharmaceutical and Clinical Research, 10(9), 219–223.

[fsn32948-bib-0064] Pereira, S. M. , Carvalho, G. Q. , Louro, I. D. , Dos Santos, D. B. , & Oliveira, A. M. (2019). Polymorphism in the vitamin D receptor gene is associated with maternal vitamin D concentration and neonatal outcomes: A Brazilian cohort study. American Journal of Human Biology, 31(4), e23250. 10.1002/ajhb.23250 31070844

[fsn32948-bib-0065] Pérez, L. F. R. , Pasupuleti, V. , Mezones‐Holguin, E. , Benites‐Zapata, V. A. , Thota, P. , Deshpande, A. , & Hernandez, A. V. (2015). Effect of vitamin D supplementation during pregnancy on maternal and neonatal outcomes: A systematic review and meta‐analysis of randomized controlled trials. Fertility and Sterility, 103(5), 1278–1288.e1274. 10.1016/j.fertnstert.2015.02.019 25813278

[fsn32948-bib-0066] Principi, N. , & Esposito, S. (2020). Vitamin D deficiency during pregnancy and autism spectrum disorders development. Frontiers in psychiatry, 10, 987. 10.3389/fpsyt.2019.00987 32082196 PMC7006052

[fsn32948-bib-0067] Purswani, J. M. , Gala, P. , Dwarkanath, P. , Larkin, H. M. , Kurpad, A. , & Mehta, S. (2017). The role of vitamin D in pre‐eclampsia: A systematic review. BMC Pregnancy and Childbirth, 17(1), 1–15. 28709403 10.1186/s12884-017-1408-3 PMC5513133

[fsn32948-bib-0068] Rajput, R. , Vohra, S. , Nanda, S. , & Rajput, M. (2019). Severe 25 (OH) vitamin‐D deficiency: A risk factor for development of gestational diabetes mellitus. Diabetes & Metabolic Syndrome: Clinical Research & Reviews, 13(2), 985–987. 10.1016/j.dsx.2019.01.004 31336556

[fsn32948-bib-0069] Ramasamy, I. (2020). Vitamin D metabolism and guidelines for vitamin D supplementation. The Clinical Biochemist Reviews, 41(3), 103–126. 33343045 10.33176/AACB-20-00006 PMC7731935

[fsn32948-bib-0070] Rosenfeld, T. , Salem, H. , Altarescu, G. , Grisaru‐Granovsky, S. , Tevet, A. , & Birk, R. (2017). Maternal–fetal vitamin D receptor polymorphisms significantly associated with preterm birth. Archives of Gynecology and Obstetrics, 296(2), 215–222. 28612095 10.1007/s00404-017-4412-y

[fsn32948-bib-0071] Roth, D. E. , Leung, M. , Mesfin, E. , Qamar, H. , Watterworth, J. , & Papp, E. (2017). Vitamin D supplementation during pregnancy: State of the evidence from a systematic review of randomised trials. BMJ, 359, j5237. 29187358 10.1136/bmj.j5237 PMC5706533

[fsn32948-bib-0072] Santamaria, C. , Bi, W. G. , Leduc, L. , Tabatabaei, N. , Jantchou, P. , Luo, Z.‐C. , Audibert, F. , Nuyt, A. M. , & Wei, S. Q. (2018). Prenatal vitamin D status and offspring's growth, adiposity and metabolic health: A systematic review and meta‐analysis. British Journal of Nutrition, 119(3), 310–319. 29321080 10.1017/S0007114517003646

[fsn32948-bib-0073] Santorelli, G. , Whitelaw, D. , Farrar, D. , West, J. , & Lawlor, D. A. (2019). Associations of maternal vitamin D, PTH and calcium with hypertensive disorders of pregnancy and associated adverse perinatal outcomes: Findings from the born in Bradford cohort study. Scientific Reports, 9(1), 1–9. 30718630 10.1038/s41598-018-37600-9 PMC6362043

[fsn32948-bib-0074] Sarma, D. , Saikia, U. K. , & Das, D. V. (2018). Fetal skeletal size and growth are relevant biometric markers in vitamin D deficient mothers: A north East India prospective cohort study. Indian Journal of Endocrinology and Metabolism, 22(2), 212–216. 29911034 10.4103/ijem.IJEM_652_17 PMC5972477

[fsn32948-bib-0075] Schutkowski, A. , Max, D. , Bönn, M. , Brandsch, C. , Grundmann, S. M. , Hirche, F. , Staege, M. S. , & Stangl, G. I. (2018). Vitamin D does not play a functional role in adipose tissue development in rodent models. Molecular Nutrition & Food Research, 62(4), 1700726. 10.1002/mnfr.201700726 29205876

[fsn32948-bib-0076] Serrano, D. N. C. , Gamboa‐Delgado, E. M. , Domínguez‐Urrego, C. L. , Vesga‐Varela, A. L. , Serrano‐Gómez, S. E. , & Quintero‐Lesmes, D. C. (2018). Vitamin D and risk of preeclampsia: A systematic review and meta‐analysis. Biomedica, 38(Suppl 1), 43–53. 10.7705/biomedica.v38i0.3683 29874709

[fsn32948-bib-0077] Shah, I. U. , Sameen, A. , Manzoor, M. F. , Ahmed, Z. , Gao, J. , Farooq, U. , Siddiqi, S. M. , Siddique, R. , Habib, A. , Sun, C. , & Siddeeg, A. (2021). Association of dietary calcium, magnesium, and vitamin D with type 2 diabetes among US adults: National health and nutrition examination survey 2007–2014—A cross‐sectional study. Food Science Nutrition, 9(3), 1480–1490. 33747462 10.1002/fsn3.2118 PMC7958525

[fsn32948-bib-0078] Shi, D. , Wang, D. , Meng, Y. , Chen, J. , Mu, G. , & Chen, W. (2021). Maternal vitamin D intake during pregnancy and risk of asthma and wheeze in children: A systematic review and meta‐analysis of observational studies. The Journal of Maternal‐Fetal & Neonatal Medicine, 34(4), 653–659. 31018731 10.1080/14767058.2019.1611771

[fsn32948-bib-0079] Simner, C. L. , Ashley, B. , Cooper, C. , Harvey, N. C. , Lewis, R. M. , & Cleal, J. K. (2020). Investigating a suitable model for the study of vitamin D mediated regulation of human placental gene expression. The Journal of Steroid Biochemistry and Molecular Biology, 199, 105576. 31904414 10.1016/j.jsbmb.2019.105576 PMC7021509

[fsn32948-bib-0080] Sucksdorff, M. , Brown, A. S. , Chudal, R. , Surcel, H.‐M. , Hinkka‐Yli‐Salomäki, S. , Cheslack‐Postava, K. , Gyllenberg, D. , & Sourander, A. (2021). Maternal vitamin D levels and the risk of offspring attention‐deficit/hyperactivity disorder. Journal of the American Academy of Child & Adolescent Psychiatry, 60(1), 142–151. e142. 31863882 10.1016/j.jaac.2019.11.021 PMC8330061

[fsn32948-bib-0081] Thorne, L. A. L. , & Fawzi, W. W. (2012). Vitamin a and carotenoids during pregnancy and maternal, neonatal and infant health outcomes: A systematic review and meta‐analysis. Paediatric and Perinatal Epidemiology, 26 Suppl 1(0 1), 36–54. 10.1111/j.1365-3016.2012.01284.x 22742601 PMC3843354

[fsn32948-bib-0082] Traglia, M. , Windham, G. C. , Pearl, M. , Poon, V. , Eyles, D. , Jones, K. L. , Lyall, K. , Kharrazi, M. , Croen, L. A. , & Weiss, L. A. (2020). Genetic contributions to maternal and neonatal vitamin D levels. Genetics, 214(4), 1091–1102. 32047095 10.1534/genetics.119.302792 PMC7153928

[fsn32948-bib-0083] Vickers, M. H. (2014). Developmental programming and transgenerational transmission of obesity. Annals of Nutrition and Metabolism, 64(Suppl. 1), 26–34. 25059803 10.1159/000360506

[fsn32948-bib-0084] Vinkhuyzen, A. A. , Eyles, D. W. , Burne, T. H. , Blanken, L. M. , Kruithof, C. J. , Verhulst, F. , Jaddoe, V. W. , Tiemeier, H. , & McGrath, J. (2018). Gestational vitamin D deficiency and autism‐related traits: The generation R study. Molecular Psychiatry, 23(2), 240–246. 27895322 10.1038/mp.2016.213 PMC5554617

[fsn32948-bib-0085] Wagner, C. L. , Hollis, B. W. , Kotsa, K. , Fakhoury, H. , & Karras, S. N. (2017). Vitamin D administration during pregnancy as prevention for pregnancy, neonatal and postnatal complications. Reviews in Endocrine and Metabolic Disorders, 18(3), 307–322. 28214921 10.1007/s11154-017-9414-3

[fsn32948-bib-0086] Wang, J. , Liu, N. , Sun, W. , Chen, D. , Zhao, J. , & Zhang, W. (2018). Association between vitamin D deficiency and antepartum and postpartum depression: A systematic review and meta‐analysis of longitudinal studies. Archives of Gynecology and Obstetrics, 298(6), 1045–1059. 30264203 10.1007/s00404-018-4902-6

[fsn32948-bib-0087] Weishaar, T. , Rajan, S. , & Keller, B. (2016). Probability of vitamin D deficiency by body weight and race/ethnicity. The Journal of the American Board of Family Medicine, 29(2), 226–232. 26957379 10.3122/jabfm.2016.02.150251

[fsn32948-bib-0088] Wen, J. , Hong, Q. , Wang, X. , Zhu, L. , Wu, T. , Xu, P. , Fu, Z. , You, L. , Wang, X. , Ji, C. , & Ji, C. (2018). The effect of maternal vitamin D deficiency during pregnancy on body fat and adipogenesis in rat offspring. Scientific Reports, 8(1), 1–8. 29321608 10.1038/s41598-017-18770-4 PMC5762667

[fsn32948-bib-0089] Woo, J. , Giurgescu, C. , & Wagner, C. L. (2019). Evidence of an association between vitamin D deficiency and preterm birth and preeclampsia: A critical review. Journal of Midwifery & Women's Health, 64(5), 613–629. 10.1111/jmwh.13014 31411387

[fsn32948-bib-0090] Workalemahu, T. , Badon, S. E. , Dishi‐Galitzky, M. , Qiu, C. , Williams, M. A. , Sorensen, T. , & Enquobahrie, D. A. (2017). Placental genetic variations in vitamin D metabolism and birthweight. Placenta, 50, 78–83. 28161065 10.1016/j.placenta.2016.12.028 PMC5319727

[fsn32948-bib-0091] Yadama, A. P. , Mirzakhani, H. , McElrath, T. F. , Litonjua, A. A. , & Weiss, S. T. (2020). Transcriptome analysis of early pregnancy vitamin D status and spontaneous preterm birth. PLoS One, 15(1), e0227193. 31995561 10.1371/journal.pone.0227193 PMC6988958

[fsn32948-bib-0092] Yan, X. , Wang, L. , Yan, C. , Zhang, X. , Hui, L. , Sheng, Q. , Hui, L. , Sheng, Q. , Xue, M. , & Yu, X. (2016). Decreased expression of the vitamin D receptor in women with recurrent pregnancy loss. Archives of Biochemistry and Biophysics, 606, 128–133. 27477959 10.1016/j.abb.2016.07.021

[fsn32948-bib-0093] Yu, L. , Guo, Y. , Ke, H.‐J. , He, Y.‐S. , Che, D. , & Wu, J.‐L. (2019). Vitamin D status in pregnant women in southern China and risk of preterm birth: A large‐scale retrospective cohort study. Medical Science Monitor: International Medical Journal of Experimental and Clinical Research, 25, 7755–7762. 31617502 10.12659/MSM.919307 PMC6816329

[fsn32948-bib-0094] Yuan, Y. , Tai, W. , Xu, P. , Fu, Z. , Wang, X. , Long, W. , Guo, X. , Ji, C. , Zhang, L. , Zhang, Y. , & Wen, J. (2021). Association of maternal serum 25‐hydroxyvitamin D concentrations with risk of preeclampsia: A nested case‐control study and meta‐analysis. The Journal of Maternal‐Fetal & Neonatal Medicine, 34(10), 1576–1585. 31284795 10.1080/14767058.2019.1640675

